# Malignant neuroleptic syndrome following deep brain stimulation surgery: a case report

**DOI:** 10.1186/1752-1947-5-255

**Published:** 2011-06-29

**Authors:** Marios S Themistocleous, Efstathios J Boviatsis, Lampis C Stavrinou, Pantelis Stathis, Damianos E Sakas

**Affiliations:** 1 Department of Neurosurgery, University of Athens Medical School, "Evangelismos" General Hospital, 45-47 Ipsilantou Str, GR-10676, Athens, Greece; 2 Department of Neurology, 1st Hospital of Social Security Services, Zaimi Str, GR-15127, Athens, Greece; 3 Hellenic Center for Neurosurgical Research, "P.S. Kokkalis," 3 Ploutarchou Str, GR-10675, Athens, Greece

## Abstract

**Background:**

The neuroleptic malignant syndrome is an uncommon but dangerous complication characterized by hyperthermia, autonomic dysfunction, altered mental state, hemodynamic dysregulation, elevated serum creatine kinase, and rigor. It is most often caused by an adverse reaction to anti-psychotic drugs or abrupt discontinuation of neuroleptic or anti-parkinsonian agents. To the best of our knowledge, it has never been reported following the common practice of discontinuation of anti-parkinsonian drugs during the pre-operative preparation for deep brain stimulation surgery for Parkinson's disease.

**Case presentation:**

We present the first case of neuroleptic malignant syndrome associated with discontinuation of anti-parkinsonian medication prior to deep brain stimulation surgery in a 54-year-old Caucasian man.

**Conclusion:**

The characteristic neuroleptic malignant syndrome symptoms can be attributed to other, more common causes associated with deep brain stimulation treatment for Parkinson's disease, thus requiring a high index of clinical suspicion to timely establish the correct diagnosis. As more centers become eligible to perform deep brain stimulation, neurologists and neurosurgeons alike should be aware of this potentially fatal complication. Timely activation of the deep brain stimulation system may be important in accelerating the patient's recovery.

## Introduction

Neuroleptic malignant syndrome (NMS) is a rare but potentially fatal disorder, with a mortality rate between 10% and 30%. It is characterized by fever, severe rigidity, autonomic instability, and an altered level of consciousness [1]. We report a unique case of NMS following the discontinuation of anti-parkinsonian drugs during the pre-operative preparation for deep brain stimulation (DBS) surgery.

### Case presentation

A 54-year-old Caucasian man with a 14-year history of Parkinson's disease (PD) was scheduled for DBS of the sub-thalamic nucleus (STN) bilaterally. His anti-parkinsonian medication consisted of 600 mg/day levodopa, 125 mg/day carbidopa, 1200 mg/day entacapone, and 0.54 mg/day pramipexole. This regimen was discontinued 18 hours prior to the DBS procedure, according to our standard protocol, to avoid medication-induced dyskinesias during surgery and to allow for the patient to be in an off-state, thus maximizing the clinical information gained by intra-operative stimulation [2]. The procedure itself was uneventful, with implantation of the DBS electrodes in the STN bilaterally (Medtronic 3389 electrodes; Medtronic, Minneapolis, MN, USA). Three hours post-operatively the patient developed tremor, muscle rigidity, and high fever resistant to common anti-pyretic drugs (paracetamol 1 g). The tremor and rigidity were attributed to PD and 200 mg levodopa three times daily was administered through a nasogastric tube. Systemic and central nervous system infection were also considered in the differential diagnosis; however, cerebrospinal fluid analysis, a chest X-ray, and blood and urine cultures were all within normal limits. A subsequent brain magnetic resonance imaging scan depicted no intracranial pathology and confirmed optimal lead placement. Twelve hours postoperatively the patient's PD features worsened: He developed severe axial and appendicular rigidity, coarse resting tremor, and prolonged spasms of the extremities.

His temperature had risen to 40°C, and his blood pressure had increased to 165/94 mmHg. A new laboratory investigation showed leukocytosis (leukocyte count, 19.4 × 10^9^/L with a shift to the left (neutrophil count, 167 × 10^9^/l). His serum levels of creatine kinase (CK) were markedly elevated to 1500 U/l with a normal CK-MB fraction, and his cardiac troponin levels were normal, indicating that the CK elevation was not of cardiac origin. At that point, the diagnosis of NMS was established on the basis of the clinical examination and the laboratory findings. Malignant hyperthermia was excluded after a negative caffeine-halothane contracture test [3]. The patient was intubated and transferred to the intensive care unit (ICU). Treatment by intravenous administration of 3 mg/kg/day dantrolene, 600 mg/day levodopa, and 60 mg apomorphine was initiated. After copious ICU treatment, the patient was extubated on the ninth post-operative day. Fifteen days post-operatively he still appeared lethargic and confused. The DBS device was consequently activated on the 20th post-operative day, as it was considered that it could accelerate the patient's recovery. Indeed, after DBS activation, the patient showed a good recovery pace and was discharged from the hospital on the 32nd post-operative day. At his six-month follow-up examination, he demonstrated an overall improvement of 15% in the Unified Parkinson Disease Rating Scale.

## Discussion

Discontinuation of anti-parkinsonian medication is a common practice prior to DBS surgery. It is known, however, that reduction of dopaminergic drugs can induce NMS. The pitfall lies in that all of the characteristic NMS symptoms can be attributed to other, more common causes associated with DBS performed in patients with PD, thus requiring a high index of clinical suspicion to establish the correct diagnosis in a timely manner. In particular, muscle rigidity and autonomic instability are very common in patients with PD and can be attributed to relevant drug modifications prior to surgery. Changes in cognition and delirium are also very common after DBS, especially when the STN is involved [4]. Fever and leukocytosis can be attributed to the procedure itself or to CNS infection. The activation of the DBS system was a matter of debate, as there were no similar cases reported in the literature. It was finally activated on the 20th postoperative day, after the patient had been extubated (stimulation settings were monopolar stimulation, pulse width 90 microseconds, frequency 130 Hz, amplitude 1.5 mV bilaterally, active contacts 1 and 5, right and left side, respectively). At that point, he appeared confused, drowsy, and disorganized with a very slow recovery speed. It was our impression that the activation of the DBS system significantly accelerated the patient's recovery, allowing for swift mobilization and intensive physiotherapy.

## Conclusion

This is the first case report of NMS associated with DBS. Although this complication is not directly related to the procedure itself, neurosurgeons and neurologists should be vigilant when discontinuing anti-parkinsonian medication prior to surgery. As the number of DBS procedures for PD increases and smaller neurosurgical centers become eligible to perform the procedure, the medical community should be aware of this potentially fatal complication, which can disguise itself within the clinical manifestations of the underlying pathology (PD) or the provided treatment (DBS). Should this complication arise, the optimal timing for DBS system activation remains to be established. Nonetheless, this case report supports the notion that stimulation may accelerate the patient's recovery.

## Consent

Written informed consent was obtained from the patient for publication of this case report and any accompanying images. A copy of the written consent is available for review by the Editor-in-Chief of this journal.

## Competing interests

The authors declare that they have no competing interests.

## Authors' contributions

MST contributed to the analysis and interpretation of the data and wrote the manuscript. DES was the chief surgeon and was involved in drafting the manuscript and critically revising it for important intellectual content. EJB, LCS, and PS made contributions to the conception and design of the case report. All authors read and approved the final manuscript, and all authors contributed equally to the final draft of the manuscript.
